# Pathological Femoral Shaft Fracture With McCune-Albright Syndrome With Hyperthyroidism Managed With Oral Alendronate: A Case Report

**DOI:** 10.7759/cureus.26802

**Published:** 2022-07-13

**Authors:** Kuldeep Bansal, Avijeet Prasad, Sumedha Singh, Ankita Chauhan, Shubham Singla

**Affiliations:** 1 Orthopaedics, University College of Medical Sciences, New Delhi, IND; 2 Radiodiagnosis, Institute of Medical Sciences and SUM Hospital, Bhubaneswar, IND; 3 Dermatology, University College of Medical Sciences, New Delhi, IND

**Keywords:** mccune-albright syndrome, café-au-lait spots, polyostotic fibrous dysplasia, oral alendronate, pathological femoral shaft fracture

## Abstract

McCune-Albright syndrome (MAS) is a complex endocrinopathy with polyostotic fibrous dysplasia and *café-au-lait *spots. Pathological femoral shaft fracture along with MAS is not a common occurrence. Bisphosphonates can be used for the management of pathological fractures.

A seven-year-old male child presented with pathological femoral shaft fracture with MAS (*café-au-lait *macules and hyperthyroidism). The patient was managed conservatively with closed reduction along with hip spica and oral alendronate. Fracture consolidation occurred within six to eight weeks with improvement in bone pains. Oral alendronate can be used as an adjuvant to conservative therapy for pathological fracture in MAS in children.

## Introduction

McCune-Albright syndrome (MAS) is a rare mosaic disorder that often manifests as a trio consisting of polyostotic fibrous dysplasia (FD), *café-au-lait* macules, and varied endocrine abnormalities (especially precocious puberty) [1]. FD can be monostotic or polyostotic; it can occur alone or as a component of MAS [1]. The most prevalent site of FD is the proximal femur, and the resultant fracture rates are the highest in childhood and decrease gradually with age [1]. We present the case of a seven-year-old boy with a pathological femoral shaft fracture, peculiar *café-au-lait* macules crossing the midline, and hyperthyroidism who was treated conservatively with oral alendronate.

## Case presentation

**Figure 1:**
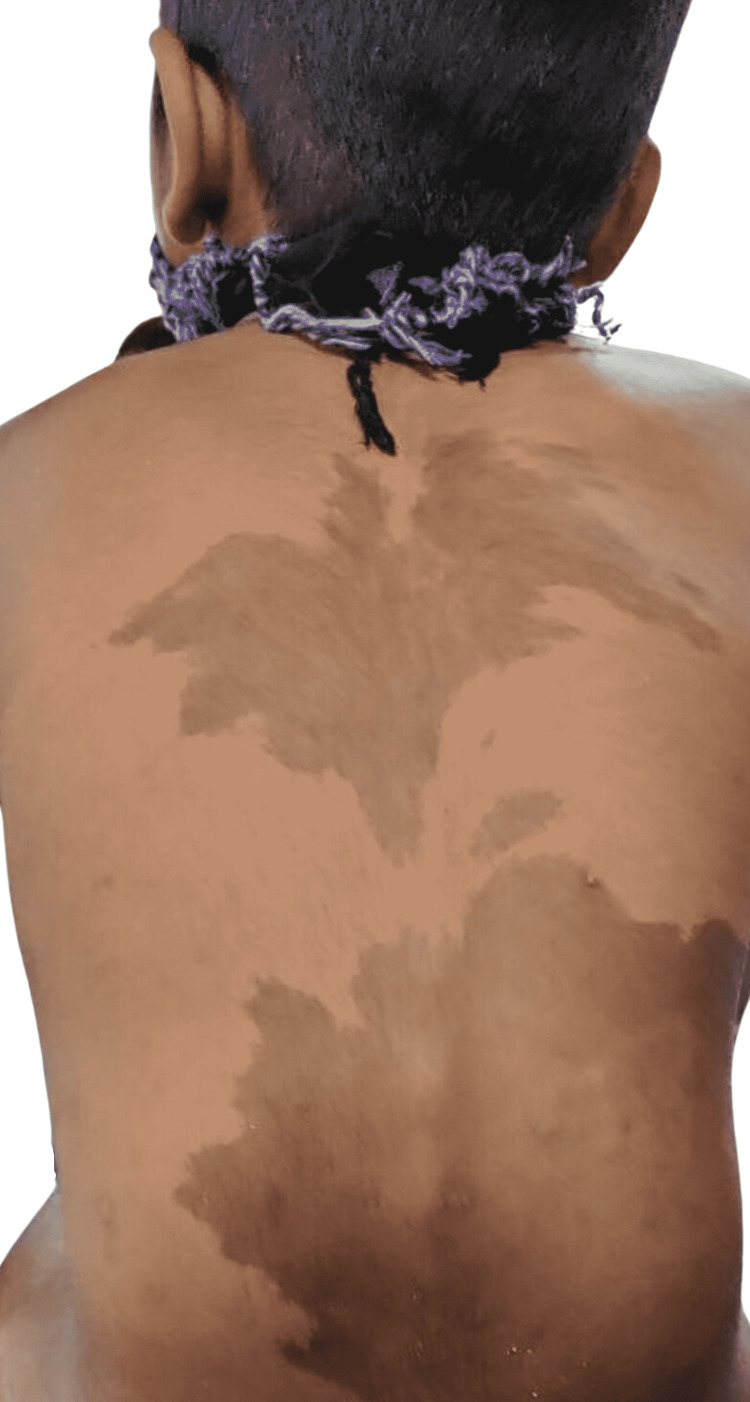
Presence of large hyperpigmented macules with irregular margins over the back and crossing the mid-line.

**Figure 2:**
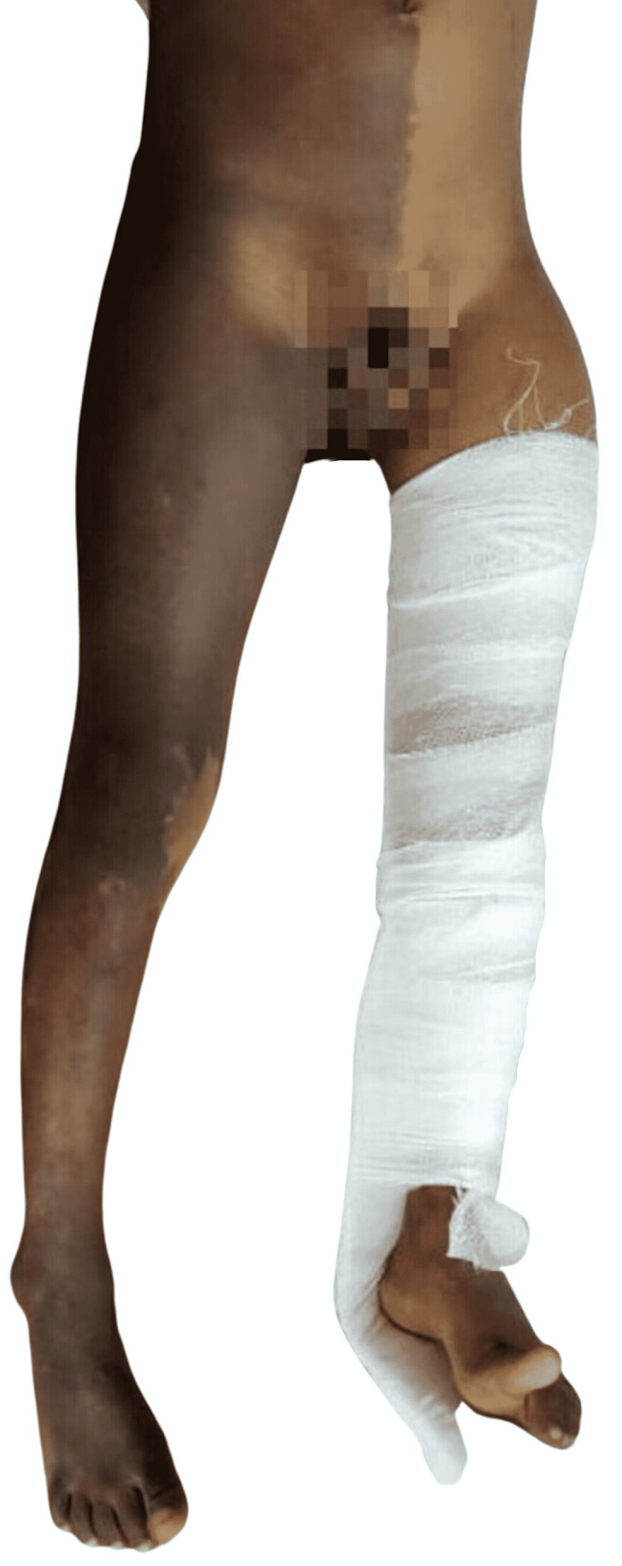
Similar macules present over the right side of the abdomen in a segmental pattern not crossing the mid-line and covering the whole of the right lower limb. Cast application can be seen on the left lower limb.

A seven-year-old boy reported to the emergency department of orthopedics with a sudden onset of pain in the left thigh and an inability to bear weight on the left lower extremity following a minor fall while playing one week prior. Considering the damage to be a mild soft tissue injury, no medical consultation was sought for the first week after the incident. As the child’s symptoms did not improve, he was brought in for evaluation. On general physical examination, the patient was lean and thin built, with three to four hyperpigmented macules on the back, abdomen (Figure 1), and right lower limb (Figure 2). The two lesions on the back were light to dark brown, ranging in size from 15 × 8 cm to 16 × 15 cm, with irregular margins (“Coast of Maine”) and crossing the midline, whereas the lesions on the abdomen and right lower limb were segmental in distribution, did not extend to the left side, and resembled *café-au-lait* spots.

**Figure 3:**
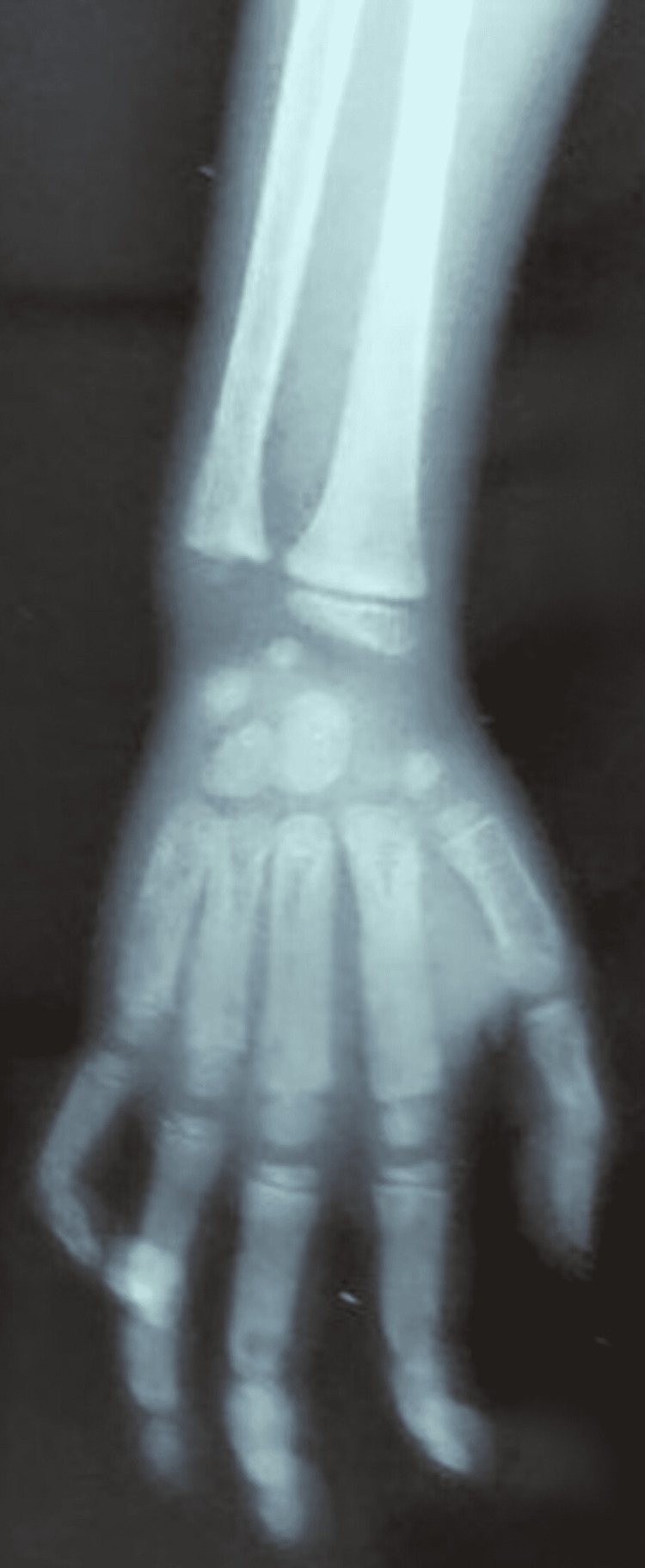
Short deformed tubular metacarpal bones.

**Figure 4:**
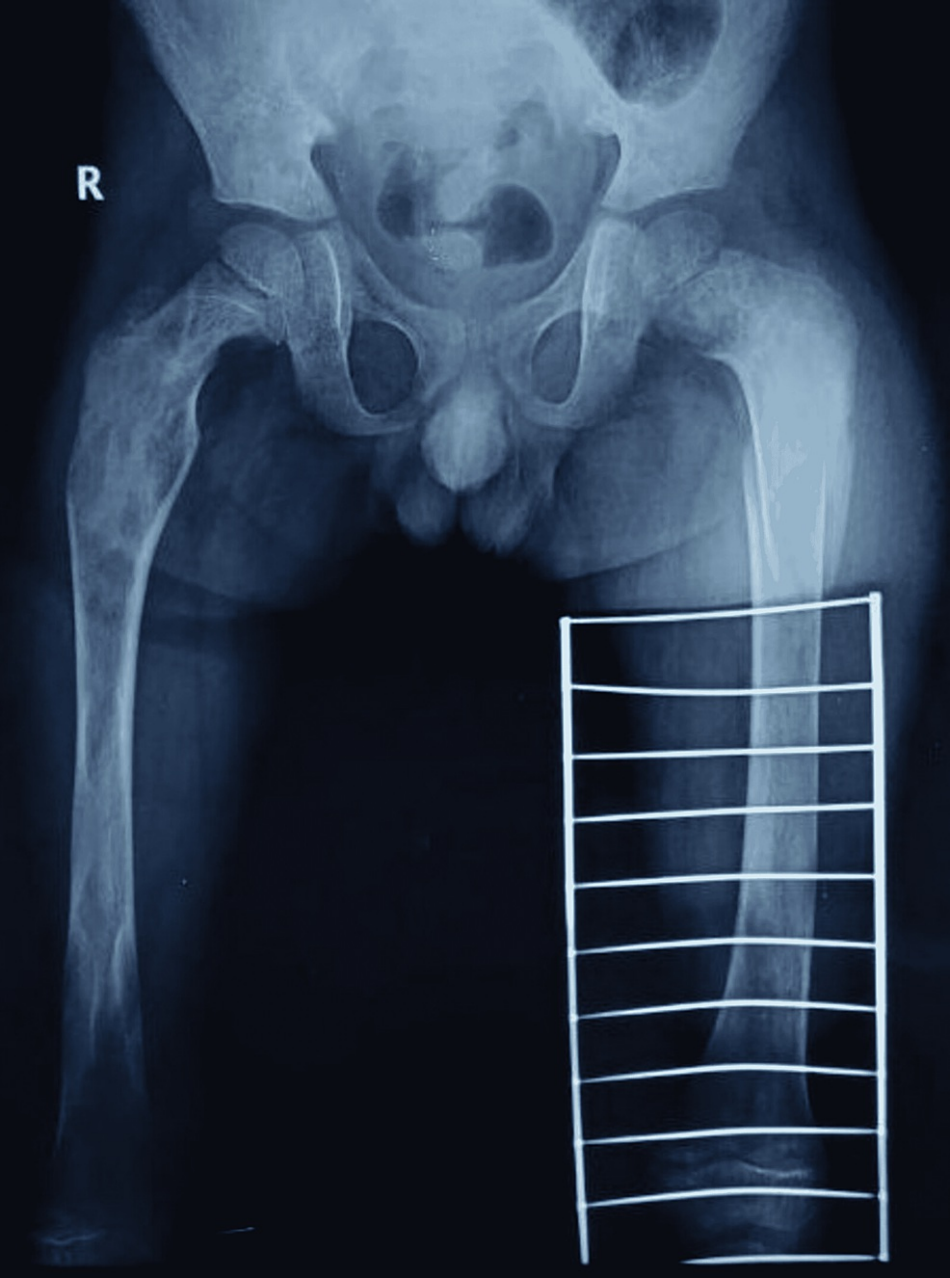
Bilateral proximal femoral deformity with a ground-glass appearance.

A plain radiograph of the patient’s left hip-thigh-knee revealed a small oblique fracture line at the junction of the upper and middle thirds of the femur, along with deformed proximal femoral anatomy with a ground-glass appearance. The case was reviewed by a multidisciplinary panel that included an orthopedist, dermatologist, radiologist, and endocrinologist. In accordance with MAS, the patient was assessed with a skeletal survey and an endocrine profile which included serum parathyroid hormone (PTH), thyroid-stimulating hormone (TSH), cortisol, and growth hormone (GH). Serum values of calcium, phosphorus, 25-OH vitamin D, and PTH were normal. Alkaline phosphatase (ALP) and 24-hour urinary hydroxyproline levels were higher, indicating increased bone turnover. The blood count, electrocardiogram, and serum liver enzymes were all within normal limits. There were no signs of precocious puberty; however, the TSH level in the serum was high. Based on a retrospective evaluation, a history of a wide-based gait with minimum to no activity restriction was obtained. The skeletal survey revealed bilateral short deformed tubular metacarpal bones (Figure 3), as well as a bilateral proximal femoral deformity with a ground-glass appearance (Figure 4), with no scoliosis or craniofacial asymmetry.

**Figure 5:**
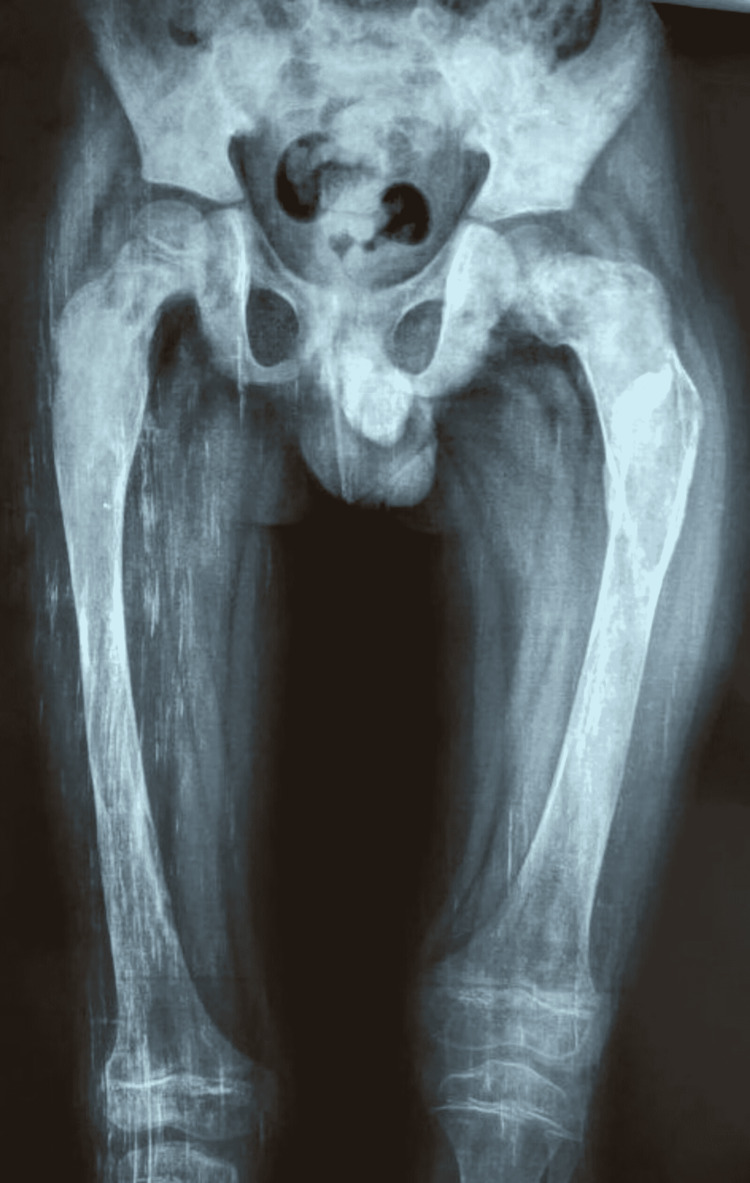
Early bridging callus across fracture at six weeks.

The objective was to fix the femoral shaft fracture following a discussion with the parents regarding the nature of the disease, but the parents refused. Consequently, the patient was treated conservatively with closed reduction and application of a hip spica, and tablet alendronate (ALN) (70 mg tablet/2 mg per kg with 200 mL of water) was administered once a week; initially, under medical supervision. After taking the drug, he was instructed to maintain fasting and an upright position for at least 30 minutes. After two weeks, the patient continued to take his prescription at home without any problems. The ALN treatment was continued for 12 weeks. At six weeks, a second X-ray revealed an early callus bridging the fracture (Figure 5).

At 12 weeks, the fracture was completely consolidated, with full knee and hip range of motion. We detected radiological thickening of the femoral shaft cortex, progressive ossification of the fibrodysplastic lesion, and periosteal bone. The parents were advised on the importance of close monitoring and follow-up for skeletal deformities.

## Discussion

MAS is a rare condition characterized by the classical triad of polyostotic FD, skin hyperpigmentation (*cafe-au-lait *patches), and endocrine abnormalities, especially precocious puberty [1]. FD is caused by activating mutations in GNAS on chromosome 3, which leads to dysregulated GαS-protein signaling in affected tissues, resulting in the replacement of healthy bone with unhealthy fibro-osseous tissue. MAS is a mosaic disorder with asymptomatic to complete disability [2-4].

For simple fractures of the shaft of the femur in children, various treatment options are available; however, with MAS, the preferred method for pathological fractures of the shaft of the femur is fixation as fracture union may be delayed in FD, and conservative treatment results in proximal femoral deformities such as coxa vara/valgus [5,6].

In this case report, because the patient’s parents refused surgery, we managed him conservatively with hip spica and oral ALN. The patient was referred to the endocrine division for additional examination and care of hyperthyroidism. Several research organizations have published case reports and patient series about the treatment of FD with bisphosphonates (pamidronate, ALN, and zoledronate). There are numerous accounts on the use of pamidronate and zolendronate [7-10]. However, to the best of our knowledge, this is the first report of FD in MAS treated with ALN. Chapurlat et al. [11] also observed that pamidronate therapy (180 mg) alleviated bone pain and reduced bone resorption with improvements in radiological results in less than half of 62 individuals treated. Liens et al. described a group of 30 patients treated with pamidronate (180 mg) at six-month intervals for three cycles, followed by annual administration [12].

Bisphosphonates administered intravenously have a satisfactory safety record overall. Hypocalcemia, as well as flu-like symptoms and brief fever, are among the adverse effects of the drug [10]. In our case, oral bisphosphonates had no side effects.

## Conclusions

Oral ALN therapy can be, in the future, considered an alternative treatment for pathological fractures in MAS because it does not alter or delay fracture healing time and reduces bone pains associated with polyostotic FD.

This case represents a child with a rare condition, MAS, with an atypical presentation of skin lesions crossing the midline on the back and being confined to the right side of the midline in the front. To our knowledge, the existence of pathological fracture in the proximal femur (FD) on the opposite side of skin involvement has not been previously recorded. For pathological fractures in MAS, an alternate therapy in the form of oral ALN and hip spica could be considered. More studies with a larger sample size need to be done to establish it as an alternative modality.
